# Loss of MED12 activates the TGFβ pathway to promote chemoresistance and replication fork stability in BRCA-deficient cells

**DOI:** 10.1093/nar/gkab1184

**Published:** 2021-12-06

**Authors:** Lindsey M Jackson, Ashna Dhoonmoon, Anastasia Hale, Kady A Dennis, Emily M Schleicher, Claudia M Nicolae, George-Lucian Moldovan

**Affiliations:** Department of Biochemistry and Molecular Biology, The Pennsylvania State University College of Medicine, Hershey, PA 17033, USA; Department of Biochemistry and Molecular Biology, The Pennsylvania State University College of Medicine, Hershey, PA 17033, USA; Department of Biochemistry and Molecular Biology, The Pennsylvania State University College of Medicine, Hershey, PA 17033, USA; Department of Biochemistry and Molecular Biology, The Pennsylvania State University College of Medicine, Hershey, PA 17033, USA; Department of Biochemistry and Molecular Biology, The Pennsylvania State University College of Medicine, Hershey, PA 17033, USA; Department of Biochemistry and Molecular Biology, The Pennsylvania State University College of Medicine, Hershey, PA 17033, USA; Department of Biochemistry and Molecular Biology, The Pennsylvania State University College of Medicine, Hershey, PA 17033, USA

## Abstract

Understanding chemoresistance mechanisms in BRCA-deficient cells will allow for identification of biomarkers for predicting tumor response to therapy, as well as the design of novel therapeutic approaches targeting this chemoresistance. Here, we show that the protein MED12, a component of the Mediator transcription regulation complex, plays an unexpected role in regulating chemosensitivity in BRCA-deficient cells. We found that loss of MED12 confers resistance to cisplatin and PARP inhibitors in both BRCA1- and BRCA2-deficient cells, which is associated with restoration of both homologous recombination and replication fork stability. Surprisingly, MED12-controlled chemosensitivity does not involve a function of the Mediator complex, but instead reflects a distinct role of MED12 in suppression of the TGFβ pathway. Importantly, we show that ectopic activation of the TGFβ pathway is enough to overcome the fork protection and DNA repair defects of BRCA-mutant cells, resulting in chemoresistance. Our work identifies the MED12-TGFβ module as an important regulator of genomic stability and chemosensitivity in BRCA-deficient cells.

## INTRODUCTION

The BRCA pathway is instrumental for genomic stability. BRCA1 and BRCA2 were initially found to play essential roles in repair of DNA double stranded breaks (DSBs) through homologous recombination (HR) (1–3). The two BRCA proteins operate in two different steps of the HR process: BRCA1 promotes DNA end resection by the CTIP nuclease at DSB sites, and BRCA2 catalyzes the loading of the RAD51 recombinase on the single stranded DNA (ssDNA) formed upon resection. This allows the subsequent strand invasion step of the HR reaction. More recently, BRCA1 and BRCA2 were also found to be essential for replication fork protection during replication stress. Upon encountering DNA lesions and other replication barriers, reversal of stalled replication forks by annealing of the two nascent strands allows stabilization of these structures (4–7). By loading RAD51 onto reversed forks, the BRCA proteins protect them against nucleolytic degradation catalyzed by nucleases such as MRE11 and DNA2 (8,9). In addition, RAD51 paralogs such as RAD51C and XRCC3 are also critical for RAD51-mediated fork protection (10,11).

In the absence of a functional BRCA pathway, the defects in DSB repair and replication fork protection result in genomic instability, underlying the tumor suppressive role of this pathway (12). Germline BRCA mutations result in increased risk of breast and ovarian tumors, and somatic inactivation of the BRCA genes is frequently found in sporadic tumors. At the same time, inactivation of BRCA1 or BRCA2 in cancers provides a unique therapeutic opportunity, since these tumors are inherently deficient in DNA repair and are thus hypersensitive to genotoxic chemotherapeutics such as cisplatin. In addition, BRCA-deficient cells are also hypersensitive to PARP1 inhibitors (PARPi) such as olaparib, since treatment with these agents result in accumulation of DSBs during DNA replication, which cannot be repaired in the absence of BRCA-mediated HR (13,14). Similar to cisplatin, PARPi may also induce replication stress by trapping PARP1 enzymes on DNA (15). In line with this, sensitivity to these agents sometimes correlates with replication fork degradation (16–18).

Despite the well-documented benefits of treating patients harboring BRCA-mutant tumors with genotoxic chemotherapy, acquired or innate resistance to cisplatin and PARPi is frequently observed (19–21). In recent years, significant effort has been put into understanding the mechanisms of chemoresistance in BRCA-deficient cells. Reversion of the BRCA mutation appears to be a common way for acquiring resistance (19). Another resistance mechanism involves restoration of HR-mediated DSB repair through rewiring of the complex regulatory switchboard that controls DSB repair pathway choice (22–30). Moreover, suppression of fork degradation can correlate with chemoresistance (16–18), suggesting that protecting stalled forks against nucleolytic degradation may also contribute to drug resistance.

Here, we show that the protein MED12, a component of the Mediator transcription regulation complex, plays an unexpected role in regulating chemosensitivity in BRCA-deficient cells. We found that loss of MED12 confers resistance to cisplatin and olaparib in BRCA1- and BRCA2-deficient cells, which is associated with restoration of both HR and fork protection. Surprisingly, MED12-controlled chemosensitivity did not involve a function of the Mediator complex, but instead we show that loss of MED12 specifically activates the TGFβ pathway, and this activation is essential for resistance to olaparib and cisplatin, and for fork protection. Our work identified the MED12-TGFβ module as an important regulator of chemosensitivity in BRCA-deficient cells.

## MATERIALS AND METHODS

### Cell culture

HeLa, RPE1 and U2OS, and MDA-MB-436 cells were grown in Dulbecco's modified Eagle's media (DMEM). DLD-1 and PEO1 cells were grown in Roswell Park Memorial Institute (RPMI) 1640 media. Media was supplemented with 10% FBS and penicillin/streptomycin. HeLa-BRCA2^KO^ cells were generated in our laboratory (30). DLD1-BRCA2^KO^ cells (Horizon HD105-007) were obtained from Dr. Robert Brosh (National Institute on Aging, Baltimore, MD). RPE1-BRCA1^KO^ (also harboring p53 homozygous deletion) were obtained from Dr Alan D’Andrea (Dana-Farber Cancer Institute, Boston, MA) (31). U2OS cells with integrated DR-GFP reporter were obtained from Dr Jeremy Stark (City of Hope National Medical Center, Duarte, CA) (32). MDA-MB-436 cells were obtained from Dr Hong-Gang Wang (Penn State College of Medicine). PEO1 cells were obtained from Dr Benjamin Bitler (University of Colorado).

Gene knockdown was performed using Lipofectamine RNAiMAX. AllStars Negative Control siRNA (Qiagen 1027281) was used as control. The following oligonucleotide sequences (Stealth or SilencerSelect siRNA, ThermoFisher) were used: ZRANB3: TGGCAATGTAGTCTCTGCACCTATA; BRCA1: AATGAGTCCAGTTTCGTTGCCTCTG; BRCA2: GAGAGGCCTGTAAAGACCTTGAATT; RAD52: GGCCAATGAGATGTTTGGTTACAAT; MED12 #1: ID s19362; MED12 #2: ID s19364; MED13: ID s19365; CDK8: ID s2831; MED7: ID s18080; TGFBR2 #1: ID s14077; TGFBR2 #2: ID s14078.

For MED12 gene knockout, the commercially available MED12 CRISPR/Cas9 KO plasmid was used (Santa Cruz Biotechnology sc-404820). Transfected cells were FACS-sorted into 96-well plates using a BD FACSAria II instrument. Resulting colonies were screened by western blot. To re-express exogenous MED12 in the knockout cell lines, cells were infected with the lentiviral construct pLV[Exp]-Puro-CMV > hMED12 (Cyagen), constitutively expressing MED12 under the control of the CMV promoter.

Denatured whole cell extracts were prepared by boiling cells in 100mM Tris, 4% SDS, 0.5M β-mercaptoethanol. Antibodies used for Western blot were: RAD51 (Abcam ab133534), ATM (Cell Signaling Technology 2873S), MED12 (Santa Cruz Biotechnology sc-515695, and Cell Signaling Technology 4529S), phospho-SMAD2 Ser465/Ser467 (Cell Signaling Technology 18338T), TGFBR2 (Cell Signaling Technology 79424S), MED7 (Abcam ab187146), MED13 (Invitrogen PA5-35924), CDK8 (ProteinTech 220671AP), ZRANB3 (Invitrogen PA5-65143), GAPDH (Santa Cruz Biotechnology sc-47724), Vinculin (Santa Cruz Biotechnology sc-73614), BRCA1 (Santa Cruz Biotechnology sc-6954), BRCA2 (Calbiochem OP95), TONSL (Invitrogen MA5-27769), RAD52 (Santa Cruz Biotechnology sc-365341), phospho-ATM Ser1981 (Cell Signaling Technology 4526S), SMAD2 (Santa Cruz Biotechnology sc-393312). RAD51 immunofluorescence was performed as described (33), using a rabbit monoclonal antibody (Abcam ab133534). Olaparib (PARP1i) and LY2109761 (TGFβRi) were obtained from Selleck Chemicals. Recombinant TGFβ1 was obtained from SigmaMillipore.

### Drug sensitivity assays

For clonogenic survival assays, 500 siRNA-treated cells were seeded per well in six-well plates and incubated with the indicated doses of olaparib or cisplatin. Media was changed after 3 days and colonies were stained after 10–14 days. Colonies were washed with PBS, fixed with a solution of 10% methanol and 10% acetic acid, and stained with 2% crystal violet (Aqua solutions). To assess cellular viability, a luminescent ATP-based assay was performed using the CellTiterGlo reagent (Promega G7572) according to the manufacturer's instructions. Following treatment with siRNA, 1500 cells were seeded per well in 96-well plated and treated with the indicated drug doses of olaparib or cisplatin. Plates were read 3 days later. For the clonogenic and cellular viability experiments with pharmacological modulation of the TGFβ pathway, TGFβRi (20μM) or TGFβ1 (5 ng/ml) were added 24 h before treatment with cisplatin and olaparib, and added again at the same time with the drugs. Apoptosis assays were performed using the FITC Annexin V kit (Biolegend, 640906). Quantification was performed using a BD FACSCanto 10 flow cytometer.

### Functional cellular assays

For the DR-GFP homologous recombination assay (32), GFP-positive cells were detected by flow cytometry 3 days after I-SceI transfection. For the DR-GFP experiments using TGFβ pathway modulators, the TGFβ receptor inhibitor LY2109761 (8 μM) or the TGFβ1 peptide (5 ng/ml) were added 24 h before I-SceI transfection, as well as for the 3 days after transfection. For the neutral comet assay, cells were pre-treated with TGFβ1 (5 ng/ml) for 24 h. Cells were then incubated in fresh media containing the indicated concentrations of olaparib or cisplatin, as well as TGFβ1 (5ng/ml), for 24h. Cells were then processed using the Comet Assay Kit (Trevigen, 4250-050) according to the manufacturer's instructions. Olive tail moment was analyzed using CometScore 2.0. BrdU alkaline comet assay was performed as previously described (34). For cell-cycle analyses, cells were fixed in 70% ethanol and stained with propidium iodide. Cell-cycle profiles were read on a BD FACSCanto 10 instrument and analyzed using FlowJo software.

### TCGA dataset analyses

Genomic, transcriptomic, and survival data for ovarian (35) and bladder (36) cancer samples, part of The Cancer Genome Atlas (TCGA), were obtained from cBioPortal (37). Survival datasets were sorted by BRCA2-status and all BRCA2-mutant samples were used for subsequent analyses. Samples were divided into three groups based on MED12 expression status in the patient tumor samples: high (0–33rd percentile), middle (33rd–67th percentile) and low (67th–100th percentile). Mantel-Cox log ranked *t* test was used for statistical analyses of the data sets using Prism software.

### DNA fiber assays

Cells were treated with siRNA and/or drugs according to the labeling schemes presented. Cells were incubated with 100 μM IdU and 100 μM CldU as indicated. Next, cells were collected and processed using the the FiberPrep kit (Genomic Vision EXT-001) according to the manufacturer's instructions. DNA molecules were stretched onto coverslips (Genomic Vision COV-002-RUO) using the FiberComb Molecular Combing instrument (Genomic Vision MCS-001). Slides were then stained with antibodies detecting CldU (Abcam 6236), IdU (BD 347580), and DNA (Millipore Sigma MAD3034) and incubated with secondary Cy3, Cy5 or BV480-conjugated antibodies (Abcam 6946, Abcam 6565 and BD Biosciences 564879). Finally, the cells were mounted onto coverslips and imaged using a confocal microscope (Leica SP5).

### Statistical analyses

For clonogenic and cellular viability assays, the two-way ANOVA statistical test was used when multiple concentrations are shown in line graphs. For bar graphs (drug sensitivity assays where only one concentration is shown, Annexin V assays, DR-GFP assays), the *t*-test (two-tailed, unequal variance) was used. For both line and bar graphs, the results shown are from independent biological replicates. For immunofluorescence assays, the *t*-test (two-tailed, unequal variance) was used. For the DNA fiber assay and the comet assay, the Mann–Whitney statistical test was performed. For immunofluorescence, DNA fiber combing, and comet assays, results from one experiment are shown; the results were reproduced in at least one additional independent biological replicate. Statistical significance is indicated for each graph (ns = not significant, for *P* > 0.05; * for *P* ≤ 0.05; ** for *P* ≤ 0.01; *** for *P* ≤ 0.001, **** for *P* ≤ 0.0001).

## RESULTS

### Loss of MED12 in BRCA-deficient cells promotes chemoresistance

**Figure 1.**
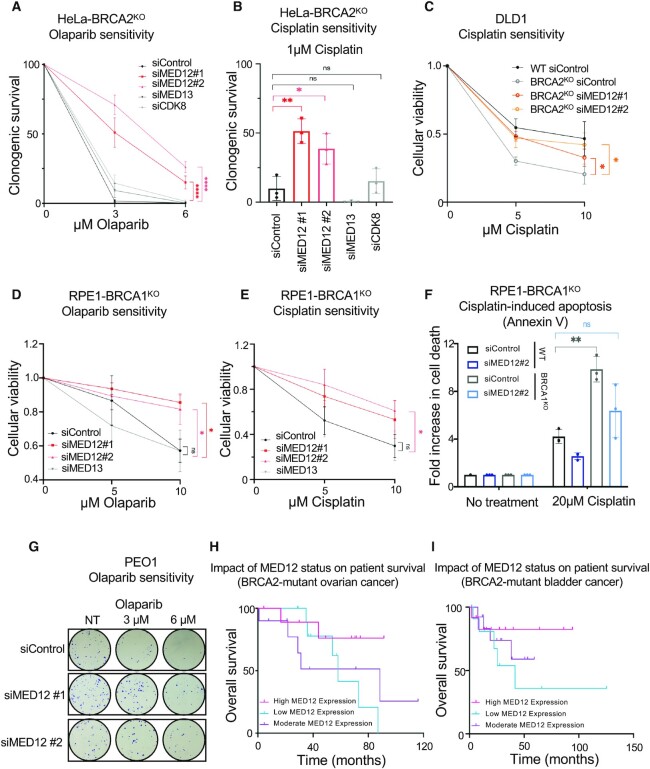
Loss of MED12 in BRCA-deficient cells promotes chemoresistance. (A, B) Clonogenic survival experiments showing that depletion of MED12, but not of MED13 or CDK8, increases the resistance of HeLa-BRCA2^KO^ cells to olaparib (**A**) and cisplatin (**B**). The average of three experiments, with standard deviations indicated as error bars, is shown. Asterisks indicate statistical significance calculated using two-way ANOVA (A) or *t*-test two-tailed, unequal variance (B). (**C**) Cellular viability assay showing that depletion of MED12 increases the resistance of DLD1-BRCA2^KO^ cells to cisplatin. The average of three experiments, with standard deviations indicated as error bars, is shown. Asterisks indicate statistical significance calculated using two-way ANOVA. (D, E) Cellular viability assay showing that depletion of MED12, but not of MED13, increases the resistance of RPE1-BRCA1^KO^ cells to olaparib (**D**) and cisplatin (**E**). The average of three experiments is presented, with standard deviations shown as error bars. Asterisks indicate statistical significance (two-way ANOVA). (**F**) Annexin V assays showing a reduction in cisplatin-induced apoptosis upon MED12 depletion in RPE-BRCA1^KO^ cells. The average of three experiments is presented, with standard deviations shown as error bars. Asterisks indicate statistical significance (t-test two-tailed, unequal variance). (**G**) Representative clonogenic assay showing that MED12 depletion in PEO1 cells promotes olaparib resistance. (H, I) Analyses of BRCA2-mutant ovarian (**H**) and bladder (**I**) TCGA cancer datasets showing that high MED12 levels are associated with increased survival, while low MED12 levels are associated with reduced survival. Mantel-Cox log ranked *t* test was used for statistical analyses (*n* = 31, *P*-value = 0.3234 for the ovarian dataset; *n* = 38, *P*-value = 0.4424 for the bladder dataset). The differences observed are not significant, likely because of the small number of BRCA2-mutant samples in the datasets.

We recently performed a genome-wide CRISPR knockout genetic screen to catalog the genetic determinants of resistance to the PARP1 inhibitor olaparib in BRCA2-deficient HeLa cells (29). Among the top hits in this screen, we noticed the presence of MED12 as a gene whose loss results in olaparib resistance (ranked 92 out of 19 112 genes present in the library employed). We thus decided to directly investigate the impact of MED12 loss on the sensitivity of BRCA-deficient cells to PARPi and cisplatin. Depletion of MED12 with two different siRNA oligonucleotides in BRCA2-knockout (BRCA2^KO^) HeLa cells resulted in significant resistance to olaparib in clonogenic assays (Figure 1A, Supplementary Figure S1A), thus validating the screen results. Moreover, MED12 depletion also restored cisplatin resistance to HeLa-BRCA2^KO^ cells (Figure 1B), as well as to DLD1-BRCA2^KO^ cells (Figure 1C, Supplementary Figure S1B).

MED12 is a component of the CDK8-module, a regulatory subunit of the Mediator transcriptional co-activator complex (38,39). However, unlike MED12 depletion, loss of other CDK8-module components such as MED13 and CDK8 did not affect the sensitivity of HeLa-BRCA2^KO^ cells to olaparib or cisplatin (Figure 1A, B, Supplementary Figure S1C, D).

Next, we investigated if the effect of MED12 depletion is restricted to BRCA2-deficient cells, or also occurs in BRCA1-deficient cells. Using cellular viability assays, we found that MED12 knockdown, but not MED13 knockdown, increased both olaparib and cisplatin resistance in BRCA1-knockout (BRCA1^KO^) RPE1 cells (Figure 1D, E, Supplementary Figure S1E, F). Moreover, MED12 depletion reduced cisplatin-induced apoptosis in these cells (Figure 1F). We also investigated the impact of MED12 loss on the chemosensitivity of cell lines derived from BRCA-mutant tumors. Similar to its impact on BRCA-knockout cells, MED12 depletion promoted olaparib and cisplatin resistance in BRCA1-mutant MDA-MB-436 breast cancer cells and BRCA2-mutant PEO1 ovarian cancer cells (Figure 1G, Supplementary Figure S2).

The findings that MED12 depletion causes resistance of BRCA-deficient cells to chemotherapeutic agents, prompted us to explore if MED12 levels have any impact on the tumor response to genotoxic chemotherapy, in clinical samples. Ovarian and bladder tumors are mainly treated with cisplatin and other DNA damaging chemotherapeutic agents (40,41). Analyses of survival and matched genotype and expression data from TCGA datasets indicated that MED12 expression can stratify the survival of individuals with BRCA2-mutant ovarian and bladder tumors: high MED12 expression trended towards increased survival, while low MED12 expression trended towards reduced survival (Figure 1H,I). This is in line with our findings that MED12 depletion causes cisplatin resistance in BRCA2-deficient cells.

In contrast to BRCA-deficient cells, MED12 depletion in wildtype (BRCA-proficient) HeLa and RPE1 cells did not significantly affect the sensitivity to cisplation or olaparib, although we did note a trend towards increased resistance (Supplementary Figure S3A–D). MED12 knockdown in wildtype HeLa, DLD1 or RPE1 cells also did not affect the protein levels of BRCA1 or BRCA2 (Supplementary Figure S3E–G). Finally, MED12 depletion did not significantly alter the cell cycle distribution of wildtype or BRCA2^KO^ HeLa cells (Supplementary Figure S4).

**Figure 2.**
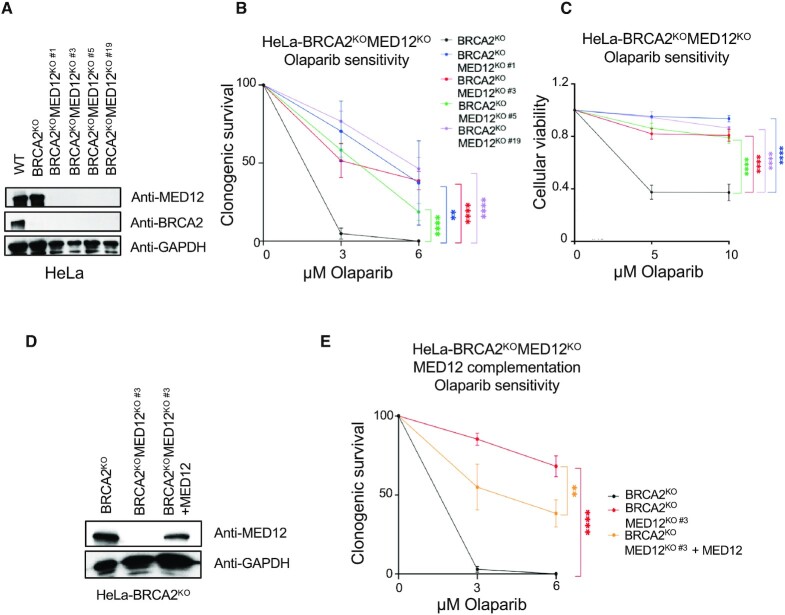
Impact of MED12-knockout on olaparib sensitivity of BRCA2-knockout cells. (**A**) Western blots confirming the loss of BRCA2 and MED12 expression in four independent double-knockout clones. (B, C) Clonogenic survival (**B**) and cellular viability (**C**) experiments showing that all four HeLa-BRCA2^KO^MED12^KO^ double knockout clones show olaparib resistance. The average of three experiments is presented, with standard deviations shown as error bars. Asterisks indicate statistical significance (two-way ANOVA). (**D**) Western blots showing the complementation of HeLa-BRCA2^KO^MED12^KO#3^ clone by exogenous re-expression of MED12. (**E**) Clonogenic survival experiments showing that complementation of HeLa-BRCA2^KO^MED12^KO^ double knockout cells by exogenous re-expression of MED12 partly restores olaparib sensitivity. The average of three experiments is presented, with standard deviations shown as error bars. Asterisks indicate statistical significance (two-way ANOVA).

To rule out potential siRNA off-target effects, we employed the CRISPR/Cas9 technology to knockout MED12 in HeLa-BRCA2^KO^ cells. We obtained four independent BRCA2-MED12 double knockout clones (HeLa-BRCA2^KO^MED12^KO^) (Figure 2A). In line with the results using siRNA-mediated MED12 depletion, all four HeLa-BRCA2^KO^MED12^KO^ cell lines showed olaparib resistance, in both clonogenic and cellular viability assays (Figure 2B, C). Moreover, we complemented one of the BRCA2-MED12 double knockout clones (HeLa-BRCA2^KO^MED12^KO#3^) by exogenous re-expression of MED12 under the control of the CMV promoter, from a lentiviral construct. Exogenous MED12 was expressed at lower levels than endogenous MED12 (Figure 2D), likely because the very large size of this protein impairs its efficient expression from the lentiviral construct. Nevertheless, the complemented cells showed partial restoration of olaparib sensitivity (Figure 2E), further ruling out non-specific effects. Altogether, these findings indicate that MED12 broadly controls the sensitivity of BRCA-deficient cells to genotoxic chemotherapeutic agents, in a manner independent of the Mediator complex.

### Activation of the TGFβ pathway underlies the chemoresistance induced by loss of MED12

**Figure 3.**
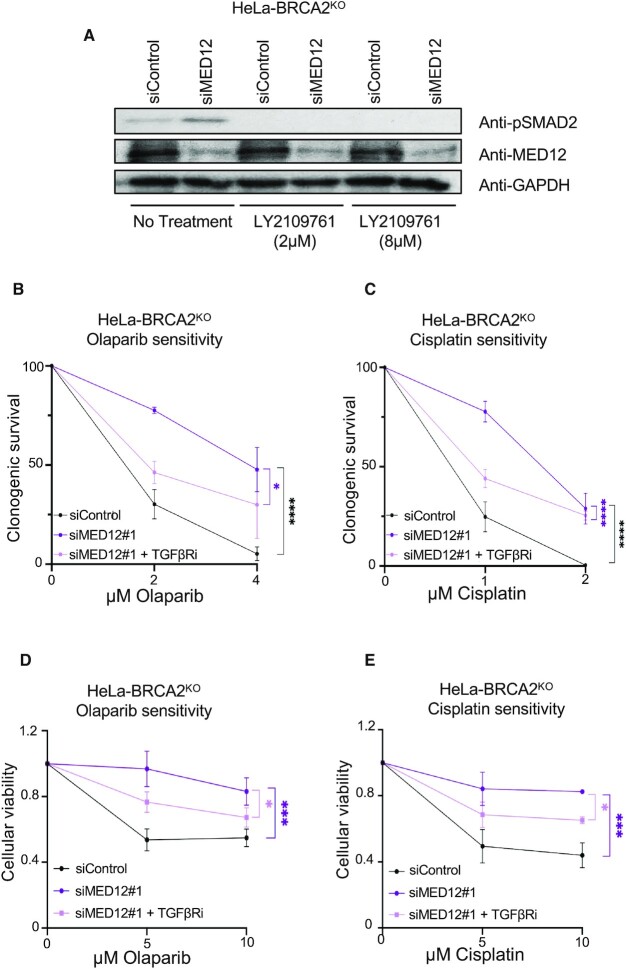
MED12 depletion promotes chemoresistance through activation of the TGFβ pathway. (**A)** Western blots showing that treatment of HeLa-BRCA2^KO^ cells with TGFβRi for 24h abolishes the SMAD2 phosphorylation induced by MED12 depletion. (B, C) Clonogenic survival experiments showing that treatment with 20 μM LY2109761 (TGFβRi) restores olaparib (**B**) and cisplatin (**C**) sensitivity in MED12-depleted HeLa-BRCA2^KO^ cells. The average of three experiments, with standard deviations indicated as error bars, is shown. Asterisks indicate statistical significance (two-way ANOVA). (D, E) Cellular viability experiments showing that treatment with 20 μM LY2109761 (TGFβRi) restores olaparib (**D**) and cisplatin (**E**) sensitivity in MED12-depleted HeLa-BRCA2^KO^ cells. The average of three experiments, with standard deviations indicated as error bars, is shown. Asterisks indicate statistical significance (2-way ANOVA).

Interestingly, loss of MED12 has been previously associated with resistance to inhibitors of ALK and EGF receptor tyrosine kinases (42). Moreover, we recently identified MED12 as a top hit in a screen designed to identify genetic determinants of the cellular response to ATR inhibitors, and showed that MED12 depletion in wildtype cells results in resistance to these inhibitors (43). In both of these cases, the impact of MED12 was shown to be independent of the Mediator complex. Instead, it involved negative regulation of the TGFβ pathway, as MED12 was shown to inhibit the glycosylation of the TGFβ receptor TGFBR2 and thereby block its expression on the cell surface. We thus tested if activation of the TGFβ pathway upon MED12 depletion is also responsible for the chemoresistance observed in BRCA-deficient cells. In line with previously-described findings (42,43), MED12 depletion in BRCA2^KO^ cells resulted in activation of the TGFβ pathway, as indicated by increased phosphorylation of SMAD2, a downstream component of the pathway (Figure 3A). Inhibition of the TGFβ pathway by treatment with the TGFβ receptor inhibitor (TGFβRi) LY2109761 abolished the increase in SMAD2 phosphorylation caused by MED12 depletion (Figure 3A). This significantly suppressed the olaparib and cisplatin resistance conferred by MED12 depletion to HeLa-BRCA2^KO^ cells, in both clonogenic and cellular viability assays (Figure 3B-E). These findings indicate that loss of MED12 in BRCA-deficient cells promotes chemoresistance, at least in part, by activating the TGFβ pathway.

### MED12 depletion restores HR in BRCA-deficient cells

**Figure 4.**
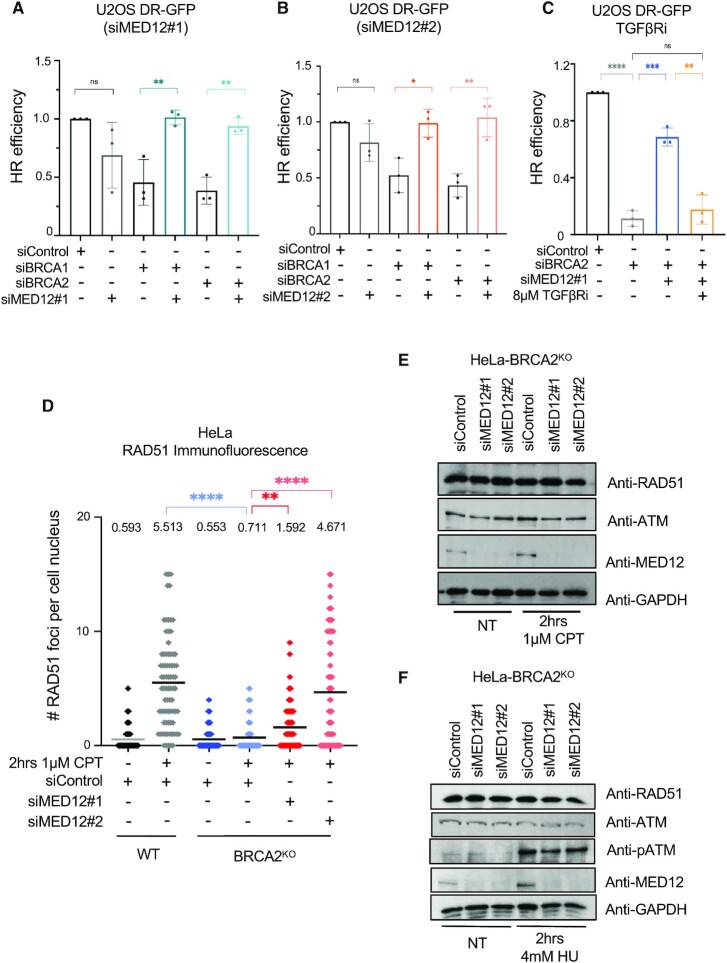
Loss of MED12 restores Homologous Recombination in BRCA-deficient cells. (**A**, **B**) DR-GFP assay showing that MED12 co-depletion with two different siRNAs restores HR efficiency in BRCA1-depleted and BRCA2-depleted cells. The average of three experiments is presented, with standard deviations shown as error bars. Asterisks indicate statistical significance (t-test two-tailed, unequal variance). (**C**) DR-GFP assay showing that TGFβ pathway inhibition with LY2109761 (TGFβRi) suppresses the restoration of HR induced by MED12 depletion in BRCA2-knockdown cells. The average of three experiments is presented, with standard deviations shown as error bars. Asterisks indicate statistical significance (t-test two-tailed, unequal variance). (**D**) RAD51 immunofluorescence experiment showing that MED12 depletion partly restores RAD51 foci formation upon CPT treatment in HeLa-BRCA2^KO^ cells. At least 75 cells were quantified for each condition. The mean value is represented on the graphs, and asterisks indicate statistical significance (t-test two-tailed, unequal variance). (E, F) Western blots showing that MED12 depletion in HeLa-BRCA2^KO^ cells does not affect the levels of RAD51 or ATM upon CPT (**E**) or HU (**F**) treatment.

We next investigated the potential mechanisms through which the loss of MED12 in BRCA-deficient cells may promote chemoresistance. Restoration of HR-mediated DSB repair has been previously associated with suppression of chemosensitivity (22–30). By employing the DR-GFP assay, which allows the measurement of HR efficiency using a reporter U2OS cell line, we found that co-depletion of MED12 reverses the HR defect caused by BRCA1- or BRCA2-knockdown (Figure 4A,B, Supplementary Figure S5). Treatment with the TGFβ receptor inhibitor LY2109761 reinstated the HR defect (Figure 4C), arguing that loss of MED12 restores HR in BRCA-deficient cells by activating the TGFβ pathway. MED12 depletion also resulted in partial restoration of RAD51 foci (which is another readout of HR efficiency) in BRCA2-knockout cells treated with the DSB-inducing agent camptothecin (CPT) (Figure 4D). Overall, these findings suggest that restoration of HR may play a role in the chemoprotective effect of MED12 depletion.

Previously, we showed that increasing RAD51 protein levels can restore HR and chemoresistance in BRCA2-deficient cells (29,30). However, loss of MED12 did not affect RAD51 levels in HeLa-BRCA2^KO^ cells treated with camptothecin or hydroxyurea (HU) (Figure 4E, F). A number of BRCA-independent mechanisms of RAD51 loading to DNA breaks have been previously described, including those governed by RAD52 and the TONSL–MMS22L complex (44,45). However, MED12 depletion did not increase the levels of RAD52 or TONSL in HeLa-BRCA2^KO^ cells (Supplementary Figure S6A, B), arguing against an involvement of these factors in HR restoration by MED12 loss in BRCA-deficient cells. In line with this, co-depletion of RAD52 did not restore olaparib sensitivity to MED12-knockdown HeLa-BRCA2^KO^ cells (Supplementary Figure S6C, D).

### The integrity of nascent DNA in BRCA-deficient cells is enhanced upon loss of MED12

**Figure 5.**
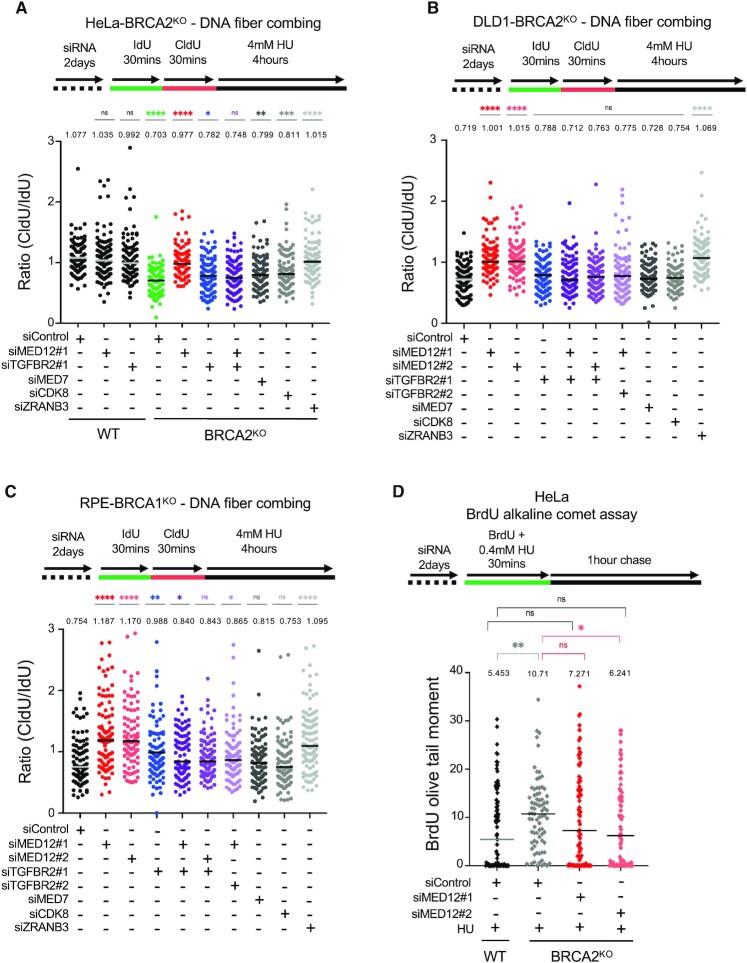
MED12 depletion promotes TGFβ-dependent fork protection in BRCA-deficient cells. (A–C**)** DNA fiber combing assays showing that MED12 knockdown, but not knockdown of MED7 or CDK8, suppresses HU-induced nascent strand degradation in HeLa-BRCA2^KO^ (**A**), DLD1-BRCA2^KO^ (**B**) and RPE1-BRCA1^KO^ (**C**) cells. Co-depletion of TGFBR2 restores fork degradation in MED12-depleted BRCA1/2-knockout cells, indicating that activation of the TGFβ pathway upon MED12 depletion is essential for fork stability. Knockdown of ZRANB3, which suppresses fork reversal, is shown as control. For all panels, the ratio of CldU to IdU tract lengths is presented, with the median values marked on the graph and listed at the top. At least 100 tracts were quantified for each sample. Asterisks indicate statistical significance (Mann-Whitney test). Schematic representations of the assay conditions are shown at the top. (**D**) BrdU alkaline comet assay showing that MED12 depletion reduces replication-associated ssDNA gaps accumulation upon HU treatment in HeLa-BRCA2^KO^ cells. At least 75 nuclei were quantified for each condition. The median values are marked on the graph and listed at the top. Asterisks indicate statistical significance (Mann-Whitney test). Schematic representations of the assay conditions are shown at the top.

Besides restoration of HR, protection of stalled replication forks against nucleolytic degradation by nucleases such as MRE11 and DNA2 was also previously proposed as a mechanism of chemoresistance in BRCA-deficient cells (16–18). We thus employed the DNA fiber combing assay to measure the impact of MED12 on replication fork stability in HeLa-BRCA2^KO^ cells. As previously described (8,9), HU treatment induces nucleolytic degradation of the nascent DNA in BRCA2-deficient cells (Figure 5A). Since only reversed forks get degraded (18,46,47), depletion of the DNA translocase ZRANB3, which abolishes fork reversal, restored fork protection (Figure 5A, Supplementary Figure S7A). Surprisingly, depletion of MED12 also restored fork protection in HeLa-BRCA2^KO^ cells to wildtype levels. In contrast, depletion of CDK8, or of another Mediator subunit, namely MED7, did not affect the nascent strand degradation observed in BRCA2-deficient cells (Figure 5A, Supplementary Figure S7B). However, co-depletion of the TGFβ receptor TGFBR2 suppressed the fork protection conferred by MED12 knockdown in BRCA2^KO^ cells (Figure 5A, Supplementary Figure S7C). These findings indicate that nascent strand protection upon loss of MED12 does not involve the Mediator complex, but instead requires TGFBR2-mediated activation of the TGFβ pathway.

To rule out a cell line-specific effect, we performed the DNA fiber analysis in additional BRCA-deficient cell lines. We obtained identical results in BRCA2-knockout DLD1 cells (Figure 5B). Moreover, similar results were obtained in RPE1-BRCA1^KO^ cells (Figure 5C, Supplementary Figure S7D–G), indicating that fork protection by MED12 loss operates in the context of BRCA1-deficiency as well.

Since, as mentioned above, fork reversal is a pre-requisite for nascent strand degradation, we investigated if fork protection upon loss of MED12 is caused by a defect in fork reversal. Fork reversal manifests as a reduction in the progression of replication forks in the presence of replication stress. To assay for this, we employed the DNA fiber assay to measure fork progression in the presence of low-dose HU (Supplementary Figure S8). As a control, ZRANB3 depletion suppressed HU-induced fork slowing, as expected from its role as a DNA translocase essential for fork reversal. In contrast, knockdown of MED12 did not affect fork reversal. These findings suggest that the effect of MED12 on nascent strand degradation does not reflect a fork reversal defect.

Recent findings identified a novel role of the BRCA pathway in suppressing accumulation of ssDNA gaps upon replication stress, (34,48–53). Using a previously-described assay for measuring ssDNA gap accumulation in newly-replicated DNA, namely the BrdU alkaline comet assay (34,54), we found that, indeed, low-dose HU treatment increased ssDNA accumulation in HeLa-BRCA2^KO^ cells compared to wildtype cells (Figure 5D). MED12 depletion partly suppressed this ssDNA accumulation, suggesting an involvement of MED12 in promoting ssDNA gap formation.

### TGFβ pathway activation in BRCA-deficient cells promotes DSB repair, replication fork stability and chemoresistance

**Figure 6.**
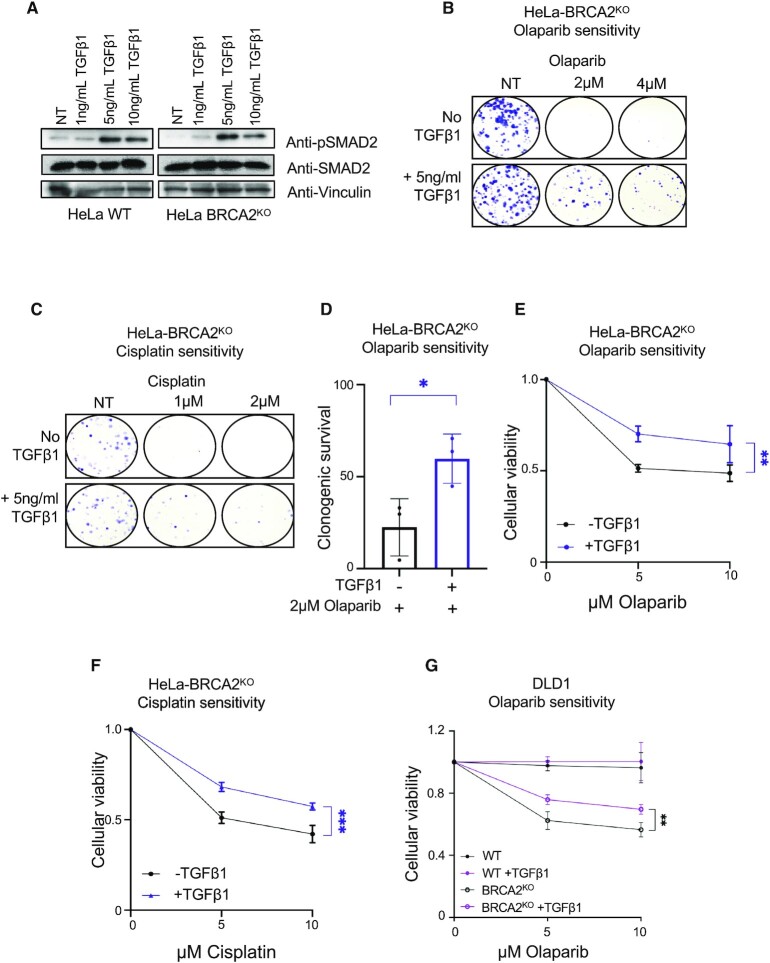
Ectopic activation of the TGFβ pathway promotes chemoresistance in BRCA-deficient cells. (**A**) Treatment of HeLa cells with recombinant TGFβ1 peptide for 24 h results in phosphorylation of SMAD2, indicating activation of the TGFβ pathway. (B, C) Representative clonogenic assays showing that treatment of HeLa-BRCA2^KO^ cells with recombinant TGFβ1 peptide promotes resistance to olaparib (**B**) and cisplatin (**C**). (**D**) Quantification of clonogenic sensitivity assays showing that TGFβ1 peptide treatment promotes olaparib resistance in HeLa-BRCA2^KO^ cells. The average of three experiments, with standard deviations indicated as error bars, is shown. Asterisks indicate statistical significance (*t*-test two-tailed, unequal variance). (E, F) Cellular viability assays showing that treatment of HeLa-BRCA2^KO^ cells with recombinant TGFβ1 peptide promotes resistance to olaparib (**E**) and cisplatin (**F**). The average of three experiments, with standard deviations indicated as error bars, is shown. Asterisks indicate statistical significance (two-way ANOVA). (**G**) Cellular viability assays showing that treatment of DLD1-BRCA2^KO^ cells with recombinant TGFβ1 peptide promotes olaparib resistance. Asterisks indicate statistical significance (two-way ANOVA).

Since activation of the TGFβ pathway appears to underlie both the chemoresistance and the fork protection observed upon loss of MED12, we sought to investigate if ectopic activation of this pathway is enough to cause chemoresistance in BRCA-deficient cells. Addition of recombinant TGFβ1 growth factor peptide to the culture media was enough to activate the TGFβ pathway in HeLa-BRCA2^KO^ cells, as demonstrated by an increase in SMAD2 phosphorylation (Figure 6A). Similar to what we showed above for MED12 depletion, treatment of HeLa-BRCA2^KO^ cells with the TGFβ1 peptide resulted in significant resistance to olaparib and cisplatin in both clonogenic and cellular viability assays (Figure 6B–F). Moreover, TGFβ1 peptide treatment also slightly increased the olaparib resistance in DLD1-BRCA2^KO^ cells (Figure 6G) and in RPE1-BRCA1^KO^ cells (Supplementary Figure S9A). A similar effect was observed when measuring olaparib and cisplatin resistance of BRCA2-mutant PEO1 ovarian cancer cells (Supplementary Figure S9B, C). In contrast, TGFβ1 peptide treatment did not impact olaparib or cisplatin sensitivity of wildtype HeLa and RPE1 cells (Supplementary Figure S10A–D), and did not affect the levels of BRCA1 and BRCA2 protein (Supplementary Figure S10E, F). These findings argue that increased TGFβ signaling is enough to promote chemoresistance in BRCA-deficient cells.

**Figure 7.**
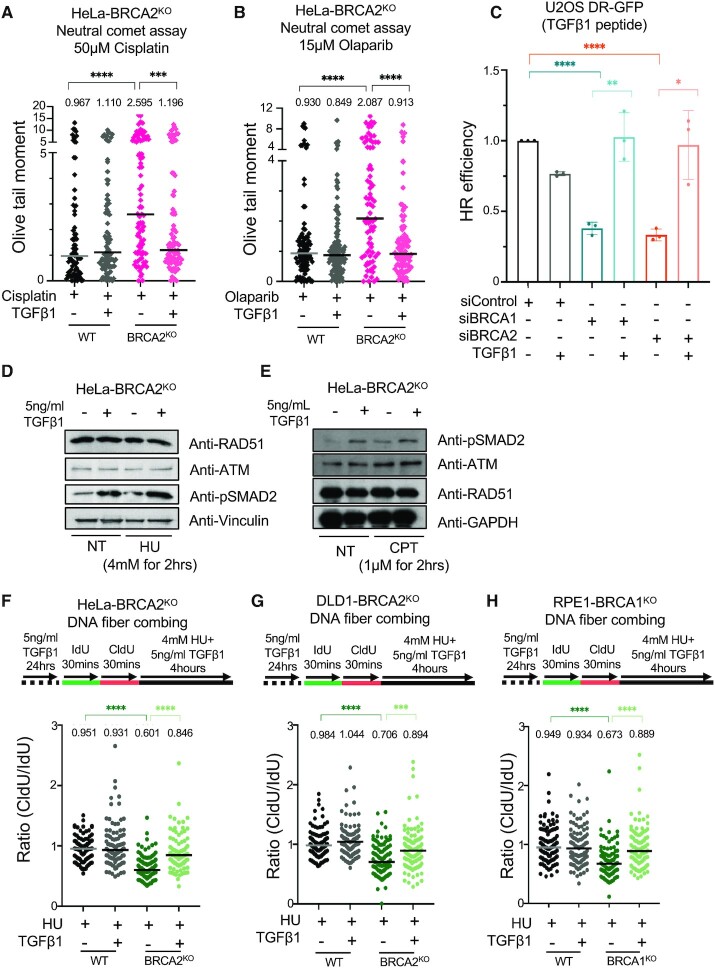
Chemoresistance upon TGFβ pathway activation in BRCA-deficient cells is associated with restoration of DSB repair and fork protection. (A, B) Neutral comet assay showing that TGFβ1 peptide treatment suppresses cisplatin (**A**) and olaparib (**B**) induced DSB accumulation in HeLa-BRCA2^KO^ cells. At least 75 comets were quantified for each sample. The median values are marked on the graph and listed at the top. Asterisks indicate statistical significance (Mann–Whitney test). (**C**) DR-GFP assay showing that TGFβ1 peptide treatment restores HR efficiency in BRCA1-depleted and BRCA2-depleted cells. The average of three experiments is presented, with standard deviations shown as error bars. Asterisks indicate statistical significance (*t*-test two-tailed, unequal variance). (D, E) Treatment of HeLa-BRCA2^KO^ cells with recombinant TGFβ1 peptide (5ng/ml for 24h) does not affect RAD51 or ATM levels upon HU (**D**) or CPT (**E**) treatment. (F–H) DNA fiber combing assays showing that TGFβ1 peptide treatment suppresses nascent strand degradation in HeLa-BRCA2^KO^ (**F**), DLD1-BRCA2^KO^ (**G**) and RPE1-BRCA1^KO^ (**H**) cells. The ratio of CldU to IdU tract lengths is presented, with the median values marked on the graph and listed at the top. At least 100 tracts were quantified for each sample. Asterisks indicate statistical significance (Mann–Whitney test). Schematic representations of the assay conditions are shown at the top.

We next investigated if chemoresistance induced by TGFβ signaling is associated with improved DNA repair. To this end, we employed the neutral comet assay to measure DSB repair. Treatment with the TGFβ1 peptide suppressed the olaparib and cisplatin-induced accumulation of DSBs in HeLa-BRCA2^KO^ cells (Figure 7A, B), indicating that activation of the TGFβ pathway promotes DSB repair in BRCA-deficient cells. In line with this, TGFβ1 treatment also restored HR efficiency as measured by the DR-GFP reporter assay (Figure 7C). Similar to MED12 depletion, this restoration of HR was not associated with increased RAD51 levels upon HU or CPT treatment (Figure 7D, E). It was recently shown that TGFβ signaling promotes the expression of ATM in K562 leukemia cells (55). However, we did not observe an impact of TGFβ1 treatment (Figure 7D, E) or of MED12 depletion (Figure 4E, F) on ATM or phosphorylated ATM levels in BRCA2-knockout HeLa cells, arguing against an ATM-mediated effect.

Finally, we investigated the impact of TGFβ pathway activation on fork protection in BRCA-deficient cells. Strikingly, treatment with the TGFβ1 peptide suppressed fork degradation in both HeLa (Figure 7F) and DLD1 (Figure 7G) BRCA2-knockout cells. Moreover, a similar restoration of fork protection was observed in BRCA1-knockout RPE1 cells (Figure 7H). These findings suggest that restoration of fork protection underlies the chemoresistance induced by TGFβ pathway activation in BRCA-deficient cells.

## DISCUSSION

Understanding the mechanisms of resistance to cisplatin and PARP inhibitors is paramount for identification of biomarkers to predict the tumor response to therapy, as well as for designing therapeutic approaches to target this chemoresistance. Here, we show that loss of MED12 promotes resistance to olaparib and cisplatin in both BRCA1- and BRCA2-deficient cells, including BRCA1- or BRCA2-knockout cells, as well as patient-derived cell lines with BRCA1 or BRCA2 mutations. This is perhaps surprising, considering the functional differences between BRCA1 and BRCA2. Nevertheless, these findings suggest that the levels of MED12 may represent a prognostic indicator of the response of BRCA-mutant tumors to genotoxic chemotherapy. Indeed, analyses of TCGA datasets of ovarian and bladder tumors indicated that MED12 expression levels can stratify the survival of patients with BRCA2-mutant tumors: low MED12 levels trended towards reduced survival of these patients, while high MED12 levels trended towards increased survival. Since chemotherapy with cisplatin and other DNA damaging agents is employed heavily for treating these types of tumors, this data suggests that low MED12 levels confer therapy resistance to BRCA-mutant tumors, thereby reducing patient survival.

Mechanistically, chemoresistance in BRCA-deficient cells has been associated with restoration of DSB repair, with protection against nucleolytic degradation of stalled replication forks, and more recently with suppression of ssDNA gap formation (16–18,22–30,34,48–53). We found that loss of MED12 in BRCA-deficient cells increases HR-mediated DSB repair, suppresses nascent strand degradation upon fork stalling, and reduces the accumulation of ssDNA gaps. The functional and mechanistic connection between these three processes is still unclear. However, it appears that they all require the activity of RAD51, which is recruited by the BRCA1-PALB2-BRCA2 complex (56). Interestingly, we noticed that MED12 depletion partly restored camptothecin-induced RAD51 foci formation in BRCA-deficient cells. We thus speculate that MED12 exerts its effect on chemosensitivity of BRCA-deficient cells by inhibiting RAD51 recruitment. How exactly this occurs remains to be discovered, since we did not observe an impact of MED12 on the levels of BRCA proteins, of RAD51 itself, or of other RAD51 loaders such as RAD52 and TONSL. Nevertheless, our findings uncovered an unexpected and multifaceted role of MED12 in DNA repair.

MED12 is a component of the CDK8-module, a regulatory sub-complex of the Mediator transcription regulation complex (38,39). Surprisingly, loss of other CDK8-module components did not phenocopy the loss of MED12, indicating that the activity of MED12 in regulating chemoresistance of BRCA-deficient cells is independent of the Mediator complex. Indeed, it was previously shown that MED12 has a separate function in regulating the expression of the TGFβ receptor TGFBR2 on the cell surface, through inhibition of TGFBR2 glycosylation which is an essential step in its maturation (42). MED12 inactivation results in increased TGFBR2 availability on the cell surface, and thus promotes the activation of the TGFβ pathway. We found that TGFβ pathway inhibition abolishes the chemoresistance and fork protection caused by loss of MED12, indicating that activation of the TGFβ pathway underlies the impact of MED12 on genomic stability in BRCA-deficient cells.

Importantly, we found that ectopic activation of the TGFβ pathway in BRCA-deficient cells was enough to restore olaparib and cisplatin resistance, efficient HR, and protection of stalled forks against nucleolytic degradation. This broad impact of TGFβ on genome stability indicates an important role in modulating DNA repair. Understanding the details of how activation of the TGFβ signaling pathway promotes genomic stability is thus of great interest. We previously showed that increasing RAD51 levels in BRCA-deficient cells is enough to restore fork protection, HR, and chemoresistance (29,30) -similar to the effect observed here upon TGFβ pathway activation. However, we did not observe an impact on RAD51 levels upon TGFβ signaling, suggesting that TGFβ-mediated chemoresistance does not involve increasing RAD51 levels.

Conflicting roles for the TGFβ pathway in DNA repair have been previously published. Activation of the TGFβ pathway was shown to result in reduced expression of ATM, BRCA1 and BRCA2 in BRCA-proficient breast cancer cells (57,58), and of RAD51 in lung epithelial cells (59) -resulting in suppression of DNA damage repair. In contrast, recent studies have shown that in BRCA-proficient leukemia cells, inhibition of the TGFβ pathway promotes a reduction in ATM, BRCA1 and BRCA2 and a concomitant increase in olaparib sensitivity (55). In our study performed in BRCA-deficient cells, we did not find an impact of TGFβ activation on ATM or RAD51 levels, perhaps reflecting cell type differences or a specific impact of BRCA deficiency. Indeed, the impact of TGFβ on DSB repair appears context-dependent. For example, a recent study found that TGFβ inhibition induces HR and suppresses non-homologous end joining (NHEJ) in hematopoietic stem cells derived from mice with Fanconi Anemia (60), while another recent study showed that both HR and NHEJ were downregulated upon TGFβ inhibition in leukemia cells (55). Thus, further work is needed to understand the cell type specificity of TGFβ-mediated genome stability.

Our work identified MED12-TGFβ as an unexpected regulatory module controlling the chemotherapy response of BRCA-deficient cells. Future studies are needed to understand the differential impact of TGFβ activation on wildtype and BRCA-deficient cells. Nevertheless, our findings suggest that direct activation of the TGFβ pathway can promote genomic stability in BRCA-deficient cells. This has potential implications not only for cancer therapy, but also for cancer prevention in BRCA-mutant individuals.

## DATA AVAILABILITY

All source data underlying each of the figures, including the values plotted in graphs, the exact *P*-values, and the uncropped blots are presented in Supplementary Table S1.

## Supplementary Material

gkab1184_Supplemental_Files
