# Supplementary material for: Loss of MED12 activates the TGFβ pathway to promote chemoresistance and replication fork stability in BRCA-deficient cells
Source: Nucleic Acids Res. 2021 Dec 6;49(22):12855–69. doi: 10.1093/nar/gkab1184 (PMC8682781; doi:10.1093/nar/gkab1184)

LEGENDS TO SUPPLEMENTARY TABLES AND FIGURES

Supplementary Table S1. The source data underlying each of the figure panels, including: the values plotted in graphs, the exact p-values, and the uncropped blots.

Supplementary Figure S1. Western blots showing: depletion of MED12 in HeLa-BRCA2^{KO} (**A**) and DLD1-BRCA2^{KO} (**B**) cells; depletion of CDK8 (**C**) and MED13 (**D**) in HeLa-BRCA2^{KO} cells; depletion of MED12 (**E**) and MED13 (**F**) in RPE1-BRCA1^{KO} cells.

Supplementary Figure S2. MED12 depletion promotes resistance to olaparib (**A, D**) and cisplatin (**B, E**) in BRCA1-mutant MDA-MB-436 breast cancer cells (**A, B**) and BRCA2-mutant PEO1 ovarian cancer cells (**D, E**). The average of three experiments, with standard deviations indicated as error bars, is shown. Asterisks indicate statistical significance (2-way ANOVA). Western blots show MED12 depletion in MDA-MB-436 (**C**) and PEO1 (**F**) cells upon siRNA treatment.

Supplementary Figure S3. Effect of MED12 depletion on olaparib (**A, C**) and cisplatin (**B, D**) sensitivity of wildtype HeLa (**A, B**) and RPE1 (**C, D**) cells. The average of three experiments, with standard deviations indicated as error bars, is shown. Asterisks indicate statistical significance calculated using t-test two-tailed, unequal variance (**A, B**) or 2-way ANOVA (**C, D**). Western blots show that MED12 depletion in HeLa (**E**), DLD1 (**F**), and RPE1 (**G**) cells does not impact the levels of BRCA1 or BRCA2.

Supplementary Figure S4. MED12 depletion does not significantly affect the cell cycle distribution of wildtype or BRCA2-knockout HeLa cells. The average of three experiments, with standard deviations indicated as error bars, is shown.

Supplementary Figure S5. Western blots showing co-depletion of MED12 with BRCA1 (**A**) and BRCA2 (**B**) in U2OS DR-GFP cells upon siRNA treatment.

Supplementary Figure S6. MED12 depletion in HeLa-BRCA2^{KO} cells does not affect the levels of RAD52 (**A**) or TONSL (**B**) proteins. Moreover, co-depletion of RAD51 (western blot shown in **C**) does not affect the olaparib resistance induced by MED12 depletion in HeLa-BRCA2^{KO} cells (**D**). The average of three experiments, with standard deviations indicated as error bars, is shown. Asterisks indicate statistical significance calculated using 2-way ANOVA.

Supplementary Figure S7. Western blots showing depletion of ZRANB3 (**A**), MED7 (**B**), and TGFBR2 (**C**) in HeLa-BRCA2^{KO} cells, and depletion of ZRANB3 (**D**), CDK8 (**E**), MED7 (**F**) and TGFBR2 (**G**) in RPE1-BRCA2^{KO} cells upon siRNA treatment.

Supplementary Figure S8. DNA fiber combing assay showing that MED12 depletion does not impact HU-induced fork slowing. Knockdown of ZRANB3, which suppresses fork reversal, is shown as control. The ratio of CldU to IdU tract lengths is presented, with the median values marked on the graph and listed at the top. At least 100 tracts were quantified for each sample. Asterisks indicate statistical significance (Mann-Whitney test). A schematic representation of the assay conditions is shown at the top.

Supplementary Figure S9. Cellular viability assays showing that TGFβ1 peptide treatment promotes olaparib resistance in RPE1-BRCA1^{KO} cells (**A**), as well as olaparib (**B**) and cisplatin (**C**) resistance in PEO1 cells. The average of three experiments, with standard deviations indicated as error bars, is shown. Asterisks indicate statistical significance (t-test two-tailed, unequal variance).

Supplementary Figure S10. Effect of TGFβ1 peptide treatment on olaparib (**A, C**) and cisplatin (**B, D**) sensitivity of wildtype HeLa (**A, B**) and RPE1 (**C, D**) cells. The average of three experiments, with standard deviations indicated as error bars, is shown. Asterisks indicate statistical significance calculated using 2-way ANOVA. Western blots show that TGFβ1 peptide treatment does not impact the levels of BRCA1 or BRCA2 in HeLa (**E**) and RPE1 (**F**) cells.

Supplementary Figure S1

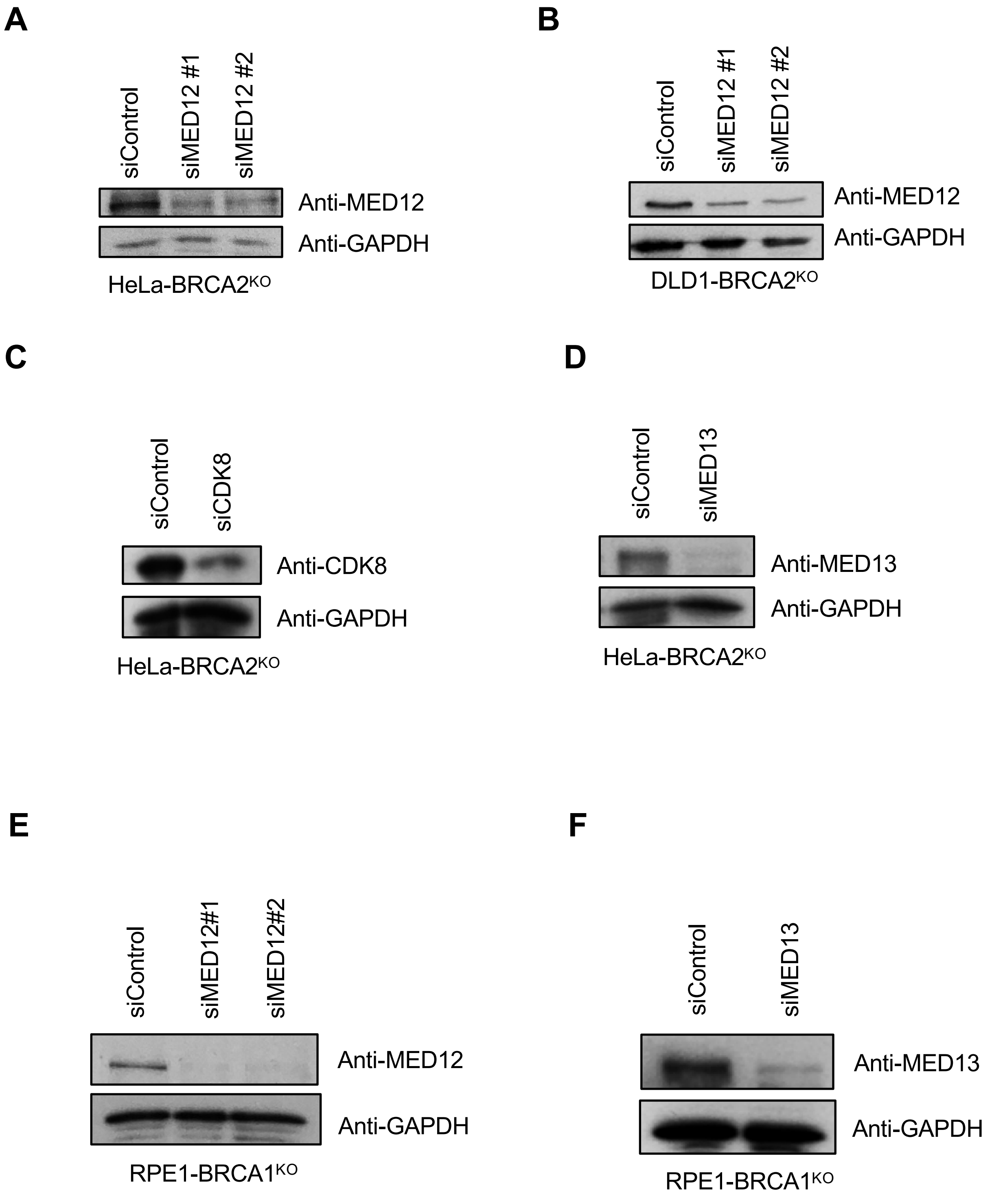

Supplementary Figure S2

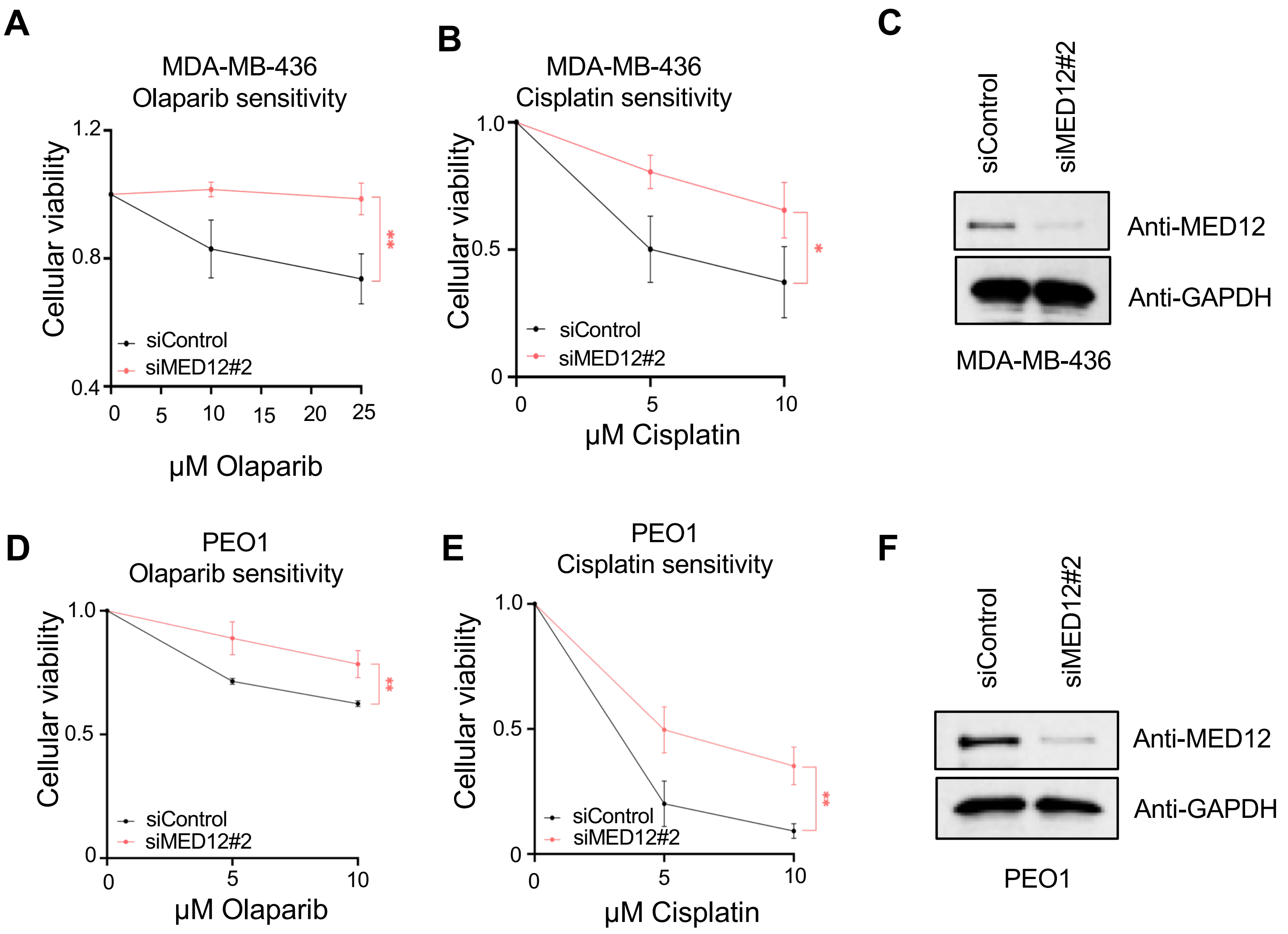

Supplementary Figure S3

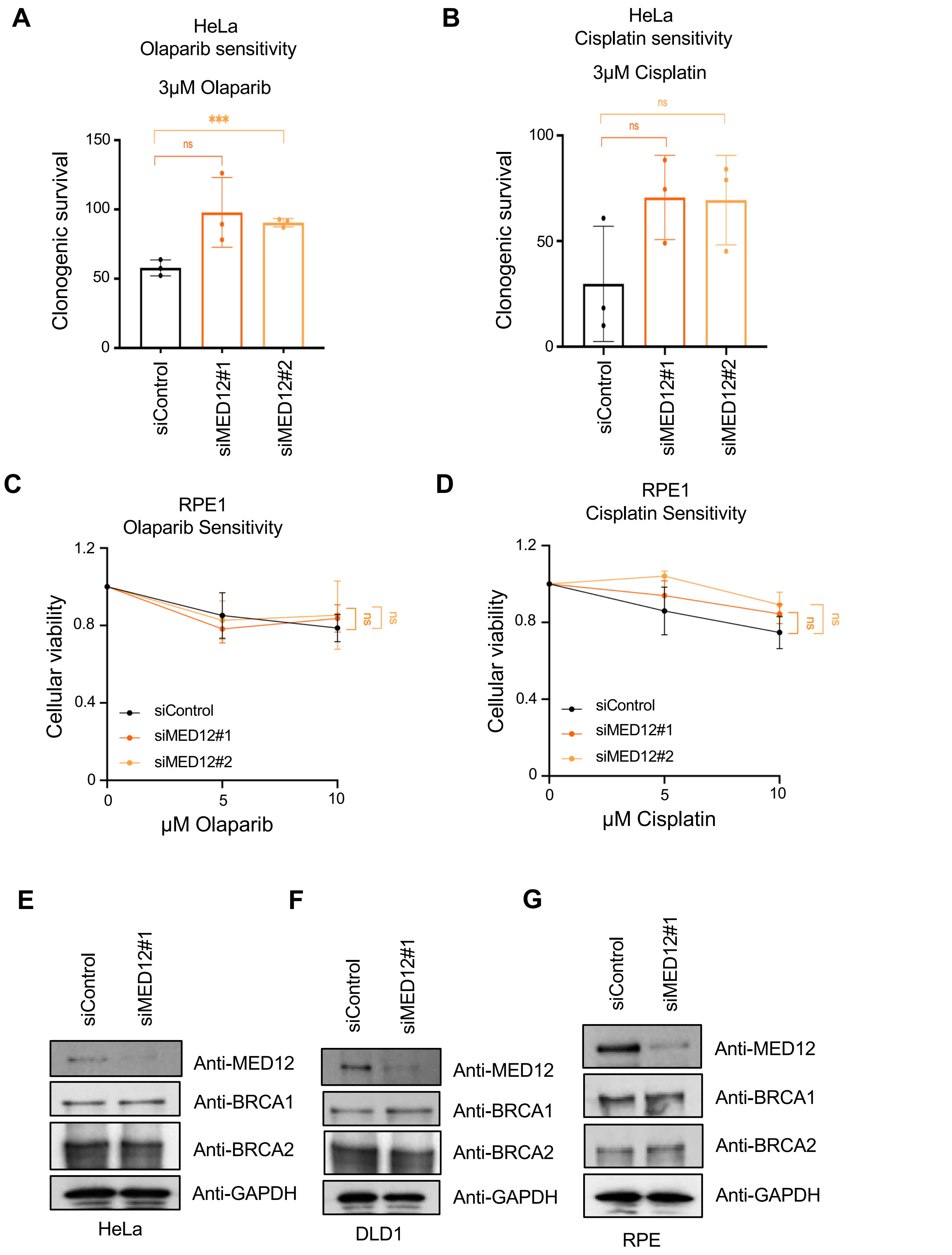

Supplementary Figure S4

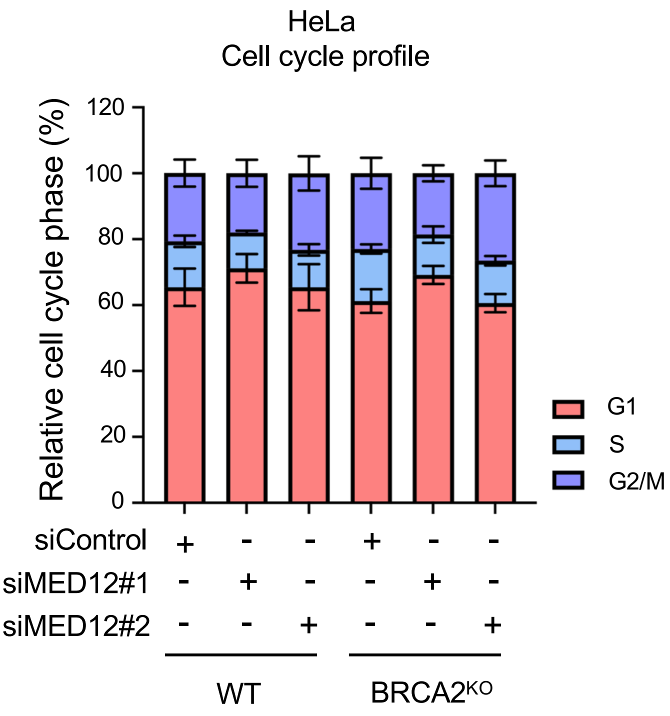

Supplementary Figure S5

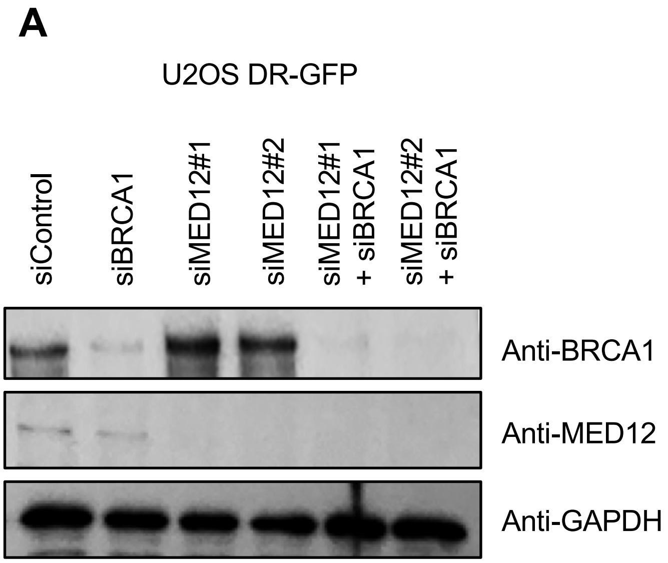

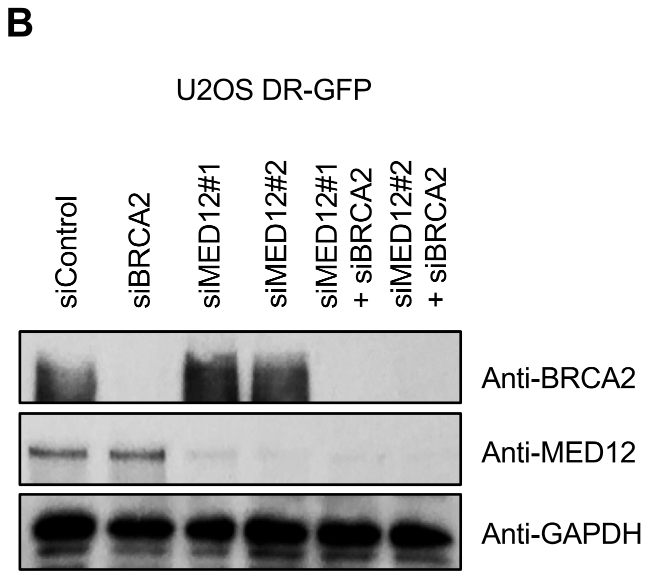

Supplementary Figure S6

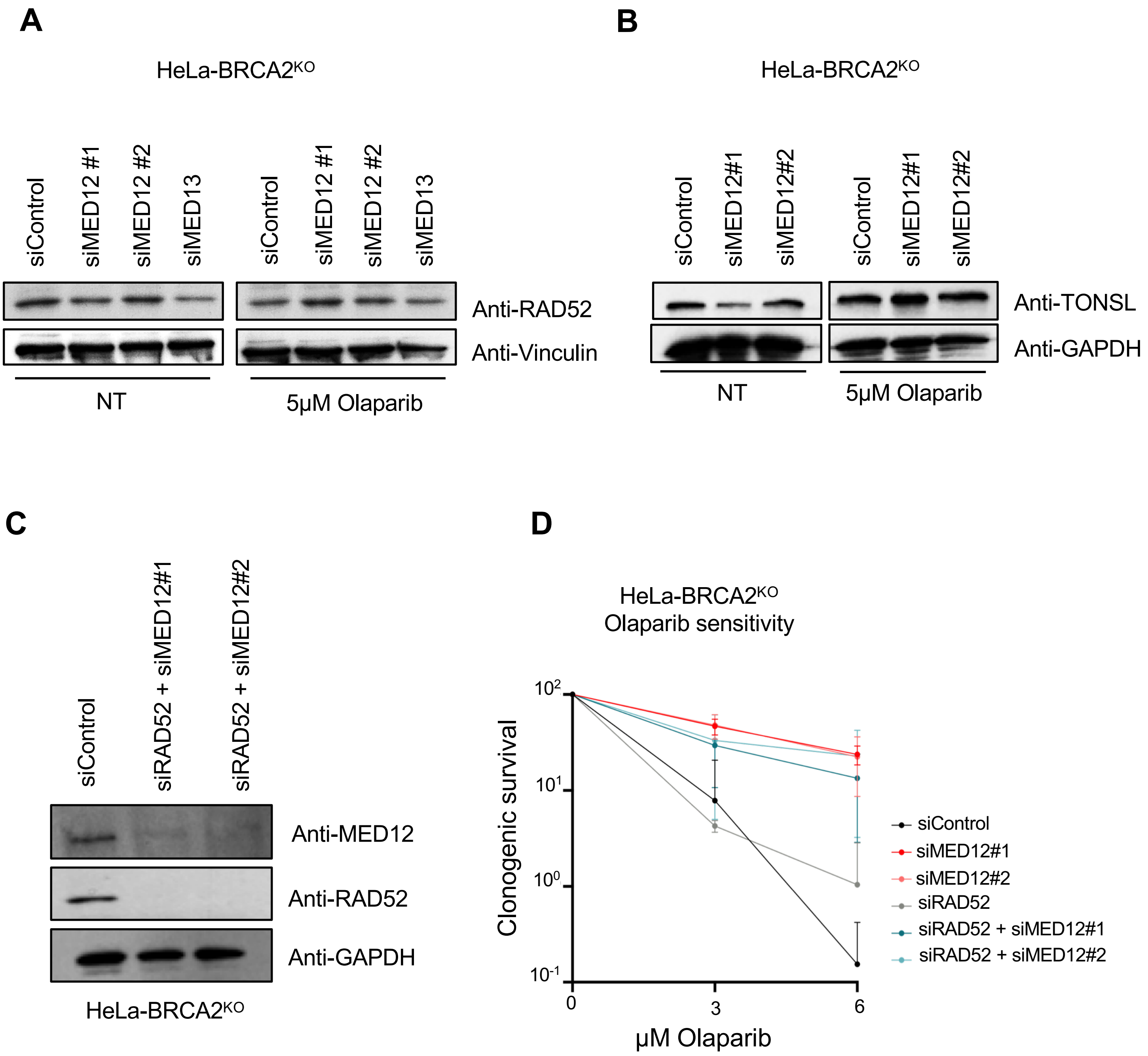

Supplementary Figure S7

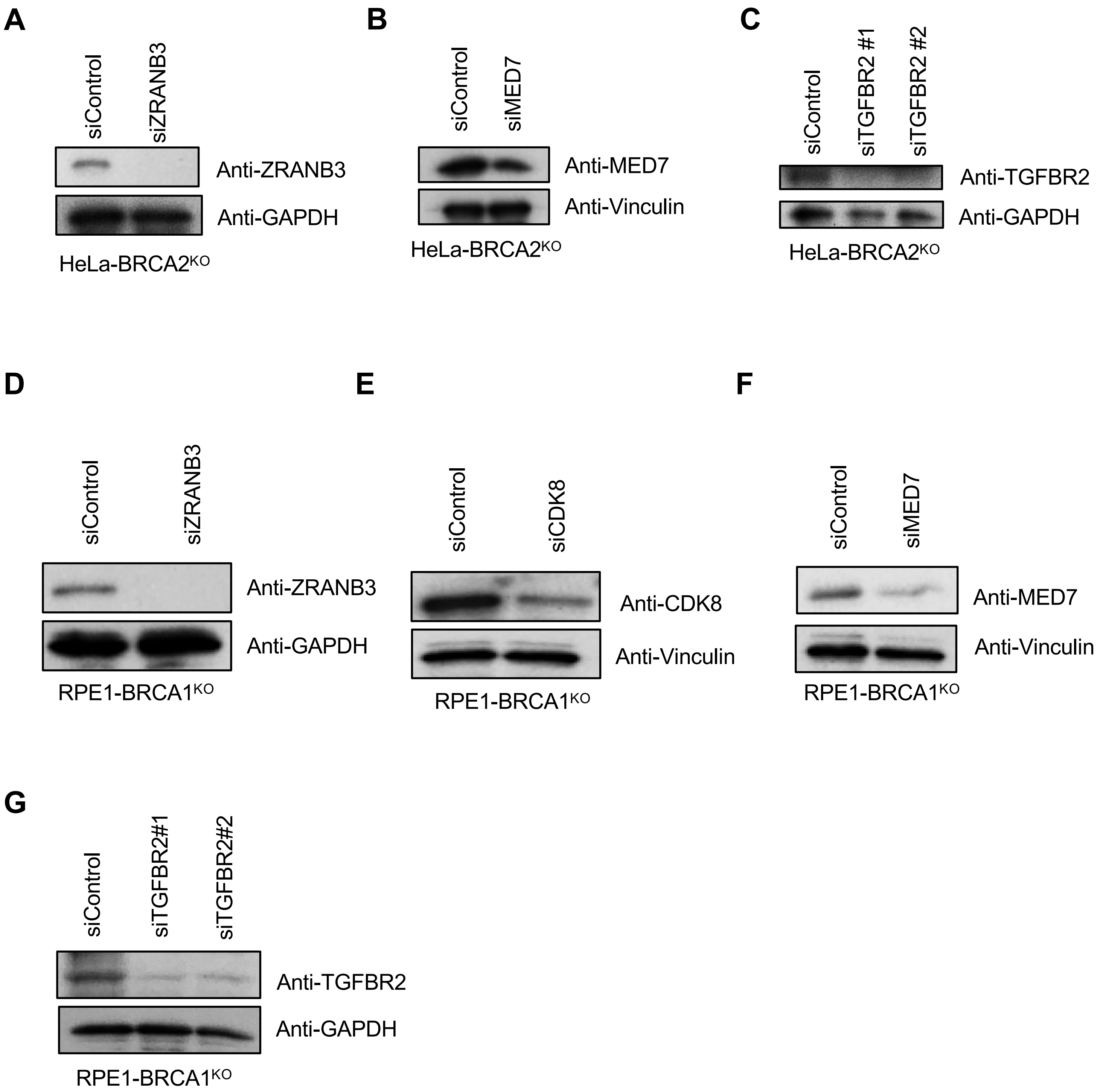

Supplementary Figure S8

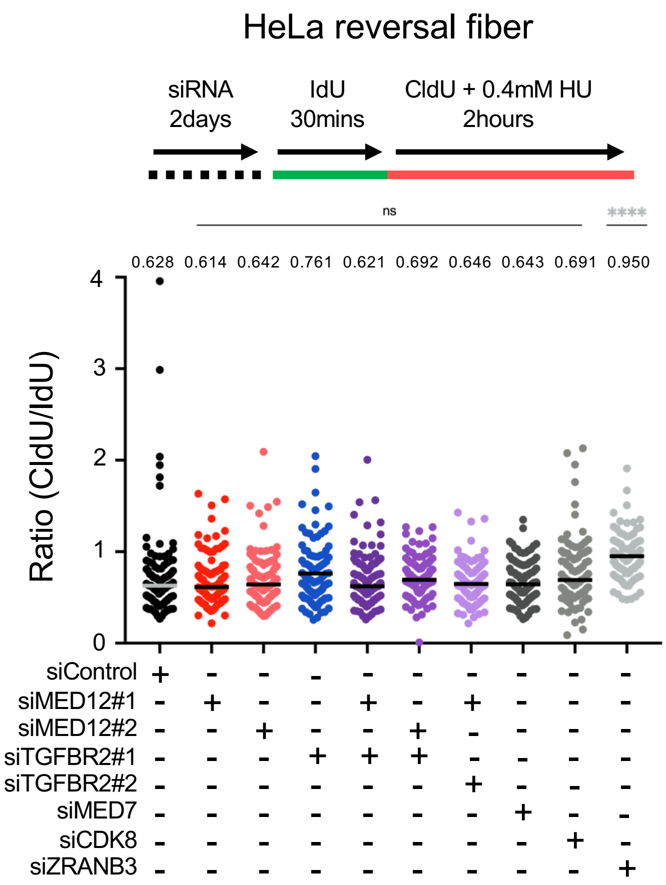

Supplementary Figure S9

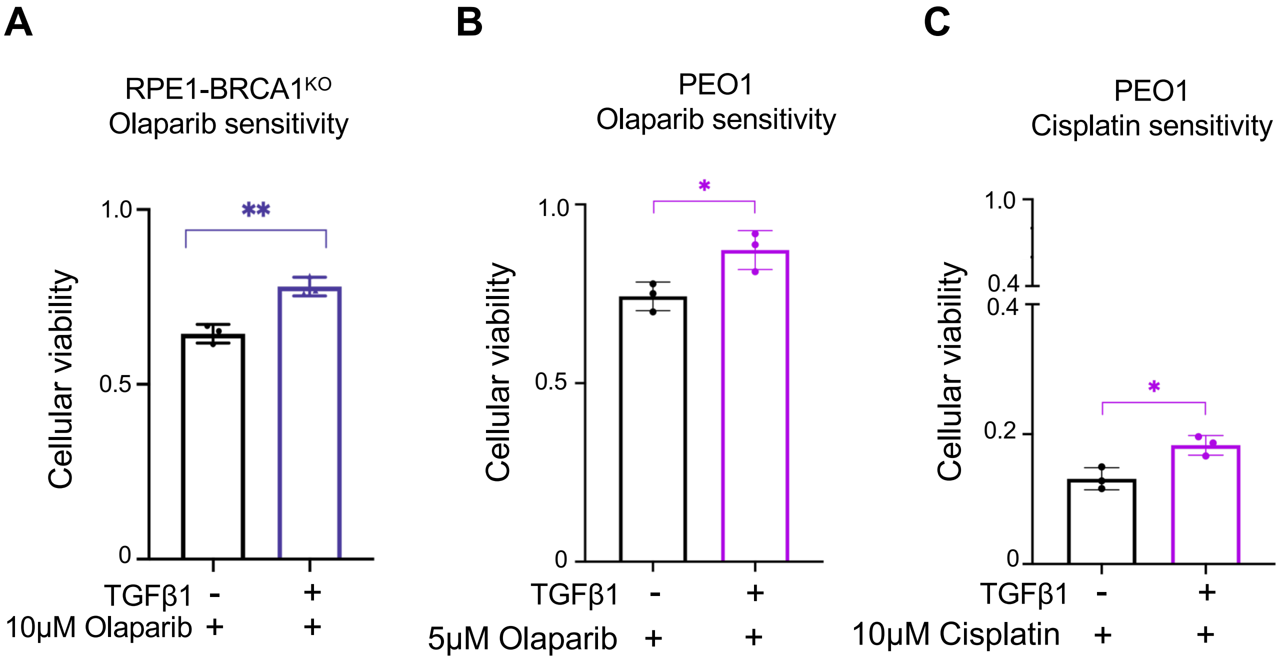

Supplementary Figure S10

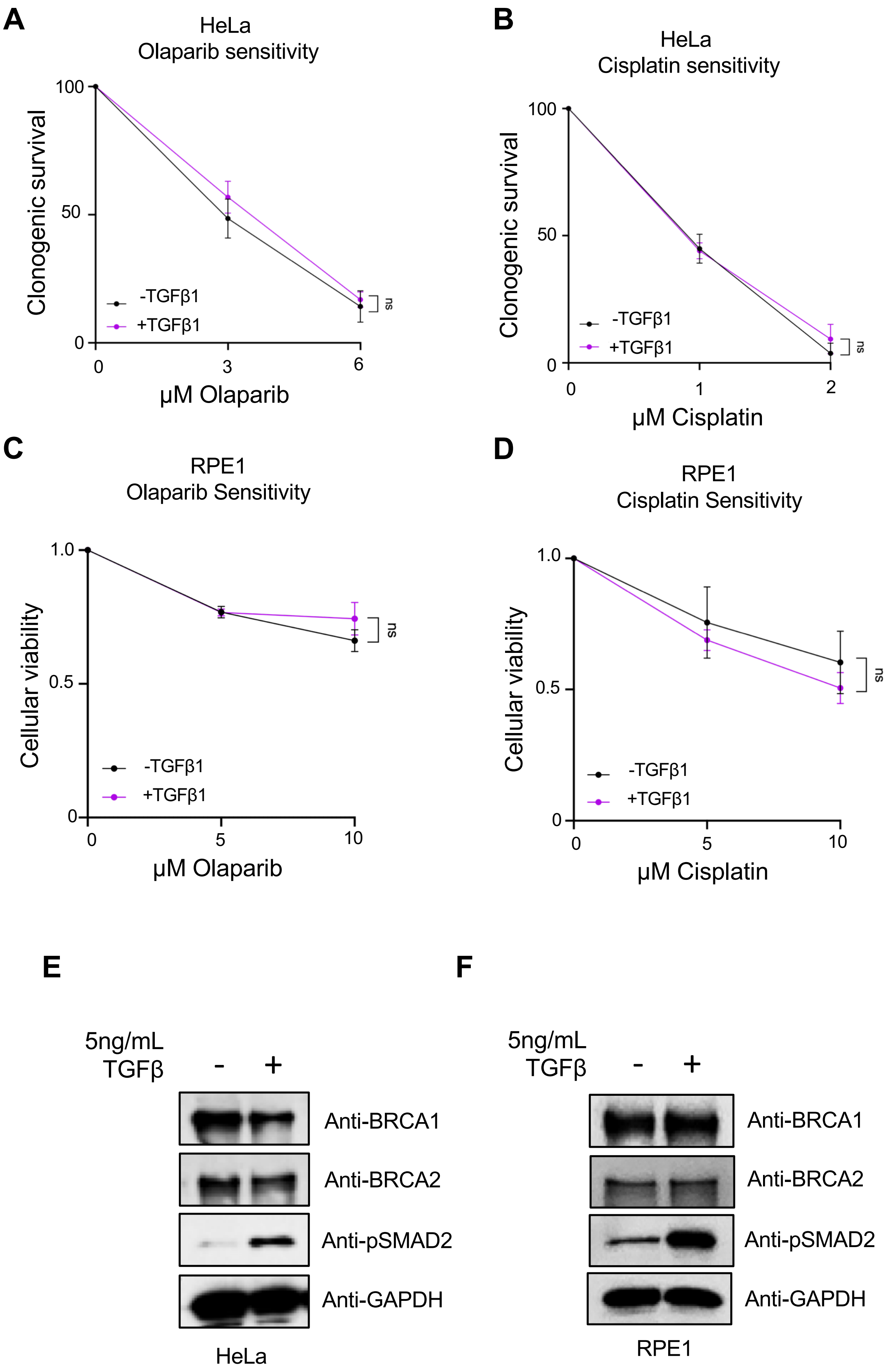

Supplement: gkab1184_Supplemental_Files [file gkab1184_supplemental_files.zip › Supplementary Material.pdf]
